# Construction of a deep - learning - based rehabilitation prediction model for lower-limb motor dysfunction after stroke using synchronous EEG-EMG and fMRI

**DOI:** 10.3389/fnins.2025.1616957

**Published:** 2025-08-21

**Authors:** Jiaqi Shi, Hongyu Wang, Haiyan Gou, Yan Chen, Jia He, Youyang Qu, Xinya Wei, Mingyue Fan, Yanlong Wang, Yanmei Zhu, Yulan Zhu

**Affiliations:** ^1^Department of Neurology, The 2nd Affiliated Hospital of Harbin Medical University, Harbin, China; ^2^Department of Neurology, The Fourth Affiliated Hospital of Harbin Medical University, Harbin, China; ^3^Department of Emergency Medicine, the First Affiliated Hospital of Harbin Medical University, Harbin, China; ^4^Department of Rehabilitation, The 2nd Affiliated Hospital of Harbin Medical University, Harbin, China

**Keywords:** rehabilitation prediction model, ischemic stroke, deep learning, model visualization, motor dysfunction

## Abstract

**Objective:**

Construct a predictive model for rehabilitation outcomes in ischemic stroke patients 3 months post-stroke using resting state functional magnetic resonance imaging (fMRI) images, as well as synchronized electroencephalography (EEG) and electromyography (EMG) time series data.

**Methods:**

A total of 102 hemiplegic patients with ischemic stroke were recruited. Resting - state functional magnetic resonance imaging (fMRI) scans were carried out on all patients and 86 of them underwent simultaneous electroencephalogram (EEG) and electromyogram (EMG) examinations. After data preprocessing, we established prediction models based on time-series data and fMRI images separately. The predictions of the time - series model and the fMRI model were integrated using ensemble learning methods to create a multimodal fusion prediction model. The accuracy, recall, precision, F1 - score, and the area under the ROC curve (AUC) were calculated to evaluate the performance of the model.

**Results:**

Compared to unimodal prediction models, multimodal fusion models demonstrated superior predictive performance. The ShuffleNet-LSTM model outperformed other multimodal fusion approaches. The area under the ROC curve was 0.8665, accuracy was 0.8031, F1-score was 0.7829, recall was 0.774, and precision was 0.833.

**Conclusion:**

A deep learning-based rehabilitation prediction model utilizing multimodal signals was successfully developed. The ShuffleNet-LSTM model exhibited excellent performance among multimodal fusion models, effectively enhancing the accuracy of predicting lower-limb motor function recovery in stroke patients.

## 1 Introduction

Stroke rehabilitation is a complex and critical research domain focused on promoting functional recovery and neural reorganization following brain injury (Kindred et al., 2019). Neuroplastic remodeling occurs not only in the acute phase following stroke but also persists throughout the rehabilitation process. It involves two principal components: functional remodeling, characterized by the redistribution of neural activity to compensate for damaged regions; and structural remodeling, encompassing neuronal regeneration, synaptic plasticity, and reorganization of neural connectivity driven by rehabilitation training (Wang et al., 2019). Recent advances in neurophysiological and imaging technologies, such as EEG, EMG, and fMRI, have enabled more precise and multidimensional investigations into the mechanisms of neural remodeling underlying post-stroke motor dysfunction. These modalities capture complementary information on neural activity, muscular responses, and cerebral hemodynamics.

With the rapid advancement of artificial intelligence (AI) technology, its application in the medical field is becoming increasingly comprehensive (Hulsen, 2022). Prognostic prediction, a critical aspect of medical treatment, has gained new opportunities through AI. By conducting in-depth analyses of patient clinical data, AI can predict disease progression and prognosis, assisting physicians in gaining a more precise understanding of patients’ conditions, formulating more effective treatment plans, and ultimately improving patient outcomes.

Ischemic stroke is associated with high incidence, mortality, and disability. Many patients experience prolonged motor dysfunction, contributing to a substantial socioeconomic burden. Consequently, accurately predicting stroke prognosis is of considerable importance. The integration of deep learning with multimodal data has emerged as a significant trend in AI research (Suzuki, 2017). Machine learning techniques can identify complex relationships among multiple variables and extract valuable insights from time-series, clinical, and imaging data. In recent years, extensive research has explored the application of machine learning in stroke studies, aiming to advance diagnosis, prognosis, and treatment strategies. Most studies have focused on using clinical data to predict outcomes after stroke (Kim et al., 2022). However, research on rehabilitation prediction incorporating multimodal data remains limited.

The deep neural network (DNN) model, a machine learning technique, is constructed within an artificial neural network (ANN) framework inspired by the structure of the human brain (Huang et al., 2022). The ANN architecture consists of multiple hidden layers positioned between the input and output layers (Du et al., 2016). In 1980, (Fukushima, 1980) the neocognitron was introduced, leading to the development of the convolutional neural network (CNN). LeCun et al. later proposed the backpropagation algorithm for training multilayer networks, significantly advancing CNN development. The LeNet-5 model achieved notable success in digital recognition tasks, marking CNN’s practical application (LeCun et al., 1998). As a representative DNN model, CNN processes two-dimensional data from multiple channels through repeated convolution and pooling operations (Hwang et al., 2021). These operations facilitate the extraction of key features from input data, enabling CNNs to identify image patterns and analyze visual information effectively. The application of deep learning algorithms to process multimodal fMRI data for developing computer-aided diagnostic and treatment tools has demonstrated significant research value and promising clinical applications. LSTM networks, (Hochreiter and Schmidhuber, 1997) a specialized form of recurrent neural networks (RNNs), are designed to handle sequence data such as time series or natural language. LSTM networks address the challenge of long-term dependency learning, which traditional RNNs struggle to achieve.

Resting-state functional magnetic resonance imaging (rs-fMRI) captures spontaneous neural activity via blood oxygen level-dependent (BOLD) signals, providing insight into large-scale functional connectivity patterns associated with post-stroke neural reorganization. Simultaneous EEG-EMG recordings offer complementary information: EEG reflects cortical excitability and functional connectivity, whereas EMG assesses peripheral neuromuscular integrity. Integrating these modalities enables a comprehensive evaluation of “cortico-muscular coupling” (Guggisberg et al., 2019).

In recent years, the integration of AI with medicine has contributed to the advancement of multimodal fusion models. Unlike traditional unimodal assessment methods, which capture only a limited aspect of a patient’s condition, multimodal fusion models integrate diverse data sources, providing a comprehensive and in-depth representation of lower limb motor dysfunction. This approach enhances the accuracy of diagnosis and supports the development of personalized treatment plans. In this study, a deep learning-based multimodal fusion prediction model was developed to assess the rehabilitation outcomes of stroke patients 3 months post-stroke.

## 2 Materials and methods

### 2.1 Data collection

#### 2.1.1 Inclusion criteria

   (1)  Diagnosis consistent with the Chinese Guidelines for the Diagnosis and Treatment of Acute Ischemic Stroke (2018).
   (2)  Age range between 40 and 79 years.
   (3)  Initial diagnosis made at least 2 weeks prior, presenting with hemiplegia and a stable condition.
   (4)  Lower limb muscle strength graded as III or higher.
   (5)  Ability to comprehend the study objectives, with informed consent obtained from the patient or family.

#### 2.1.2 Exclusion criteria

   (1)  Unstable vital signs.
   (2)  Severe diseases affecting the heart, lungs, kidneys, liver, or other organs.
   (3)  Lower limb motor dysfunction resulting from causes other than stroke.
   (4)  History of seizures or medication use affecting cortical excitability.
   (5)  Scalp or lower limb skin injuries interfering with EEG and electromyography (EMG) assessments.
   (6)  Severe cognitive impairment preventing participation in experimental tasks.

A total of 102 stroke patients were recruited from the Rehabilitation Department of the Second Affiliated Hospital of Harbin Medical University, and resting-state functional MRI (rs-fMRI) data were obtained from all participants. Among them, 86 patients additionally underwent synchronized electroencephalogram (EEG) and electromyogram (EMG) assessments at baseline to predict rehabilitation outcomes, which were categorized as either good or poor at a 3-month follow-up. Rehabilitation outcomes were evaluated using the lower-limb Fugl-Meyer Assessment (FMA) scale, which has a maximum score of 34 points. According to the 2016 American Stroke Rehabilitation Guidelines (AHA/ASA), patients with baseline lower-limb FMA scores ≤ 17 (≤50% of maximum) are classified as high-risk for persistent mobility deficits and require intensive, task-specific gait training (Winstein et al., 2016). Furthermore, multiple studies have demonstrated that an FMA score ≤ 17 predicts difficulties in regaining independent walking ability (Sullivan et al., 2010). Therefore, an FMA score of 17 was selected as the cutoff to distinguish between “good” and “poor” outcomes. All baseline assessments–including rs-fMRI, EEG-EMG, and initial FMA– were completed within 2 weeks of hospital admission. Follow-up FMA evaluations were conducted 3 months post-stroke (±1 week).

### 2.2 Clinical data collection and scale evaluation

Clinical data collection was conducted using a data acquisition system independently developed by Harbin Institute of Technology. The FMA scale assessment was performed by professional rehabilitation department personnel.

### 2.3 EEG and EMG tests

A hip/knee flexion-extension experimental paradigm was developed, with each trial comprising four sequential phases: preparation, flexion, rest, and extension. Data were collected using a synchronized EEG-EMG acquisition system independently developed by the Harbin Institute of Technology, which enables hardware-level synchronization across 16channels at a sampling rate of 1000 Hz. Electroencephalographic (EEG) signals were recorded using the international 10–20 system, with primary electrode sites including P4, CP2, FC5, C3, P3, C2, FC6, C4, CP6, F3, FC2, FC1, F4, CP5, C1, and CP1. Electromyographic (EMG) signals were obtained from major muscles involved in hip and knee movements, including the rectus femoris, vastus medialis, long head of the biceps femoris, gluteus maximus, semitendinosus, and proximal rectus femoris. Detailed recording procedures are provided in the Methods section.

### 2.4 Resting-state fMRI examination

fMRI data were acquired using a 3.0 T GE Architect superconducting MR scanner in the hospital’s magnetic resonance unit. During rs-fMRI data collection, all participants were instructed to lie flat, remain quiet, minimize movement, keep their eyes open, and avoid systematic cognitive activities. The rs-fMRI scanning parameters included a visual field of 220 mm × 220 mm, a matrix of 64 × 64 × 35, a TR of 2000 ms, a TE of 30 ms, a dynamic scan duration of 240 s, 35 layers, and a layer thickness of 4 mm.

### 2.5 Data preprocessing

Time-series data were recorded at a sampling frequency of 1000 Hz over 9 min and stored in txt format. MRI data consisted of high-resolution axial slices. To preprocess temporal data, a 150 Hz bandpass filter was applied to eliminate low-frequency drift and high-frequency noise, followed by artifact removal. The filtered signals were segmented into non-overlapping 10-s windows, with each window representing an independent input sample. To enhance model training stability, each signal window was normalized to zero mean and unit variance. For MRI data preprocessing, the images were resampled to a uniform resolution, and non-informative slices were removed through a slice screening process. Each remaining MRI slice was resized to 224 × 224 pixels for input into the deep learning model. All preprocessing steps were implemented using a standardized Python data processing pipeline to ensure consistency. Resting-state fMRI data were available for 102 patients, among whom 86 also had synchronized EEG and EMG recordings. A stratified random sampling method was employed to ensure that the proportion of patients with “good” and “poor” recovery outcomes was maintained across all data subsets. For the fMRI-based models (*n* = 102), the data were split into 70% for training (*n* = 71), 15% for validation (*n* = 15), and 15% for testing (*n* = 16). For the EEG-EMG-based models (*n* = 86), 70% were used for training (*n* = 60), 15% for validation (*n* = 13), and 15% for testing (*n* = 13).

### 2.6 Rehabilitation prediction model construction

#### 2.6.1 Predictive model based on temporal sequence data

To extract temporal features from EEG and EMG time-series data, a deep learning-based temporal model was developed. The model architecture comprised three layers of stacked LSTM units, with hidden layer sizes of 128, 64, and 32 units, respectively. A fully connected layer was applied at the final stage to complete the binary classification task, outputting the probability of rehabilitation outcomes. Cross-entropy loss was utilized as the loss function, and the Adam optimizer was employed with an initial learning rate of 0.001. A dynamic learning rate adjustment mechanism was incorporated to enhance convergence efficiency during training.

#### 2.6.2 Predictive model based on MRI imaging

A 2D convolutional neural network (2D-CNN) was constructed for MRI image-based prediction. MRI slices from each patient were treated as independent input samples. The model architecture was built upon ResNet50, FBNet, GhostNet, RegNet, and ShuffleNet. Transfer learning was employed by initializing the network with pre-trained weights, followed by fine-tuning on the study dataset to optimize performance for the rehabilitation prediction task. For each MRI slice, the network outputted a probability value corresponding to the predicted rehabilitation outcome.

#### 2.6.3 Multimodal fusion predictive model

The multimodal fusion model integrates prediction outputs from the temporal sequence model and the MRI-based model using an ensemble learning approach. The prediction scores derived from the temporal data model are averaged to obtain a temporal feature prediction score. This score is then combined with the prediction score from the MRI model to generate the final rehabilitation outcome prediction. Weight coefficients are optimized through cross-validation to ensure effective integration of information from different modalities.

### 2.7 Experimental setting

All models were trained on NVIDIA RTX 3090 GPUs with batch sizes of 32 for temporal sequence data and 16 for MRI slice data. The training process spanned 50 epochs, with the learning rate dynamically adjusted after each epoch based on validation set loss. An early stopping strategy was employed to mitigate overfitting. Additionally, online data augmentation techniques were applied to MRI data, including random rotation (−15° to 15°), horizontal flipping, and brightness adjustments, to enhance the model’s robustness.

### 2.8 Model evaluation

To assess model performance, key evaluation metrics including accuracy, recall, precision, F1-score, and the area under the ROC curve (AUC) were computed. The predictive performance of the multimodal fusion model was compared against single-modality models to demonstrate improvements in accuracy and robustness.

### 2.9 Model interpretability

To enhance the clinical interpretability of the model, Gradient-weighted Class Activation Mapping (Grad-CAM) was used to visualize prediction results for MRI images. Grad-CAM generates heatmaps highlighting the most significant activation regions contributing to classification outcomes. Specifically, Grad-CAM calculates the gradient of classification results on the final network feature map through backpropagation, utilizing gradient-weighted feature maps to indicate areas of high model attention. The generated heatmaps were overlaid onto original MRI slices to visually identify critical brain regions that influenced rehabilitation predictions.

## 3 Results

In the imaging group, FBNet demonstrated the highest performance across all evaluation indicators, exhibiting superior classification capability. The validation set achieved an accuracy of 0.7138, with notable advantages in recall rate, F1-score, and precision. In contrast, ResNet50 showed the weakest performance among the models compared (Table 1).

**TABLE 1:** fMRI prediction model evaluation.

Method	Accuracy	Recall	F1-score	Precision
FBNet	0.7138	0.7416	0.7132	0.7524
GhostNet	0.6338	0.6275	0.6264	0.6259
Regnet	0.6123	0.6667	0.5917	0.7595
Shufflenet	0.7169	0.6896	0.6928	0.7191
Resnet50	0.5815	0.5	0.3677	0.2908

In the EEG-EMG group, classification analysis of temporal data using the LSTM model resulted in a validation set accuracy of 0.68, a recall of 0.6833, an F1-score of 0.6753, and a precision of 0.6763 (Table 2).

**TABLE 2:** EEG - EMG prediction model evaluation.

Method	Accuracy	Recall	F1-score	Precision
LSTM	0.68	0.6833	0.6753	0.6763

In the multimodal fusion group, classification performance improved significantly by integrating the outputs of the image model and the LSTM sequence model. The FBNet_LSTM fusion model achieved a validation accuracy of 0.8 with an F1-score of 0.7741, ranking as the second-best among the fusion models. However, further improvements were observed with the ShuffleNet_LSTM fusion model, which attained an accuracy of 0.8031 and an F1-score of 0.7829, demonstrating the effectiveness of multimodal fusion in enhancing information complementarity (Table 3).

**TABLE 3:** Multimodal fusion prediction model evaluation.

Method	Accuracy	Recall	F1-score	Precision
FBNet_LSTM	0.8	0.7652	0.7741	0.8507
GhostNet_LSTM	0.7754	0.7368	0.7426	0.8308
Regnet_LSTM	0.76	0.7287	0.7343	0.7819
Shufflenet_LSTM	0.8031	0.774	0.7829	0.833
Resnet50_LSTM	0.6615	0.709	0.6508	0.7764

**FIGURE 1:**
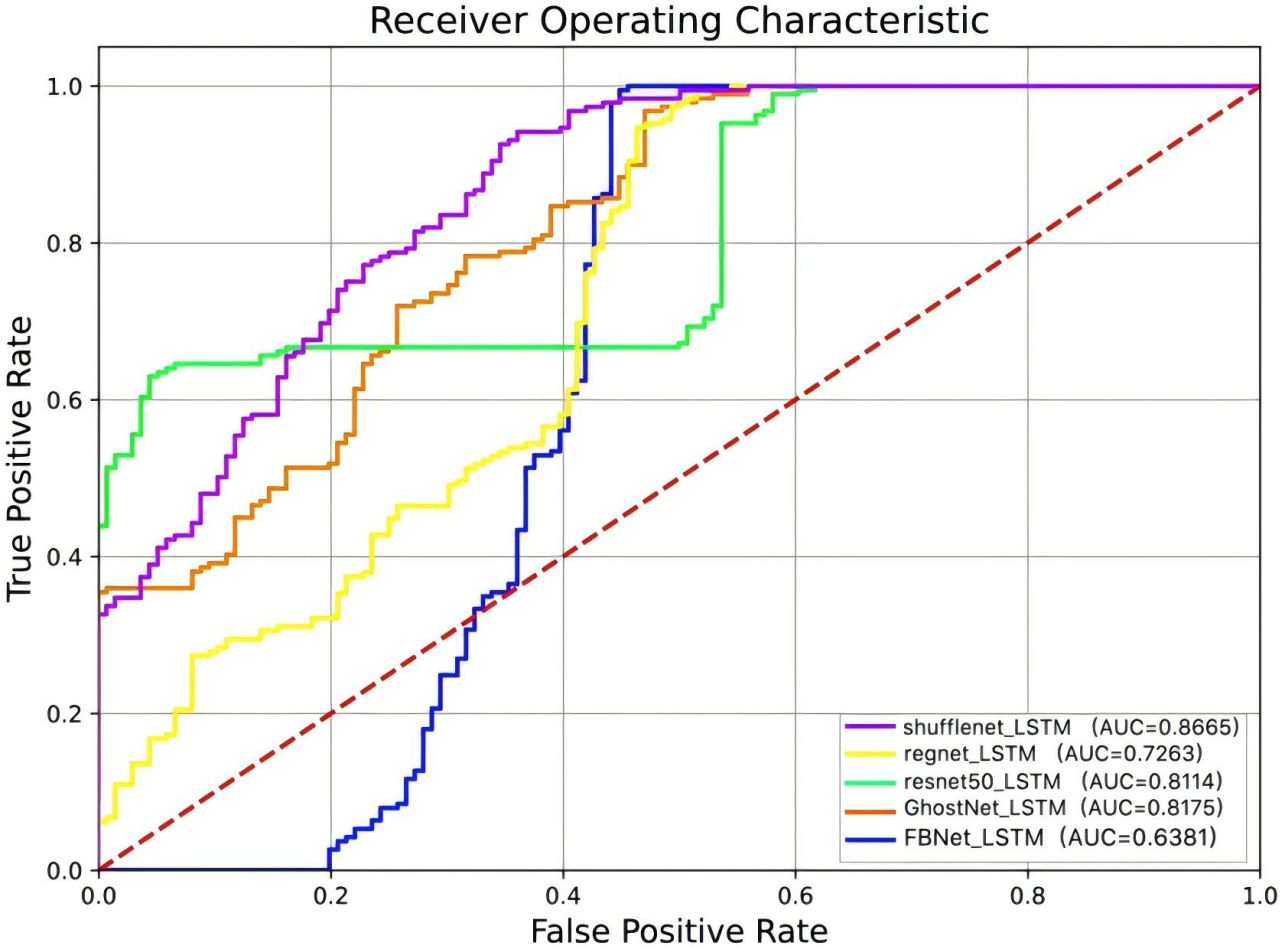
ROC curves of the multimodal fusion models.

ROC curves serve as an effective tool for evaluating classification model performance, particularly in threshold selection and addressing class imbalance. These curves illustrate model performance by depicting variations in true positive and false positive rates across different thresholds. The area AUC is utilized to quantify the overall classifier performance. For the ShuffleNet_LSTM model, the ROC curve exhibits an AUC of 0.8665, indicating strong classification capability (Figure 1).

**FIGURE 2:**
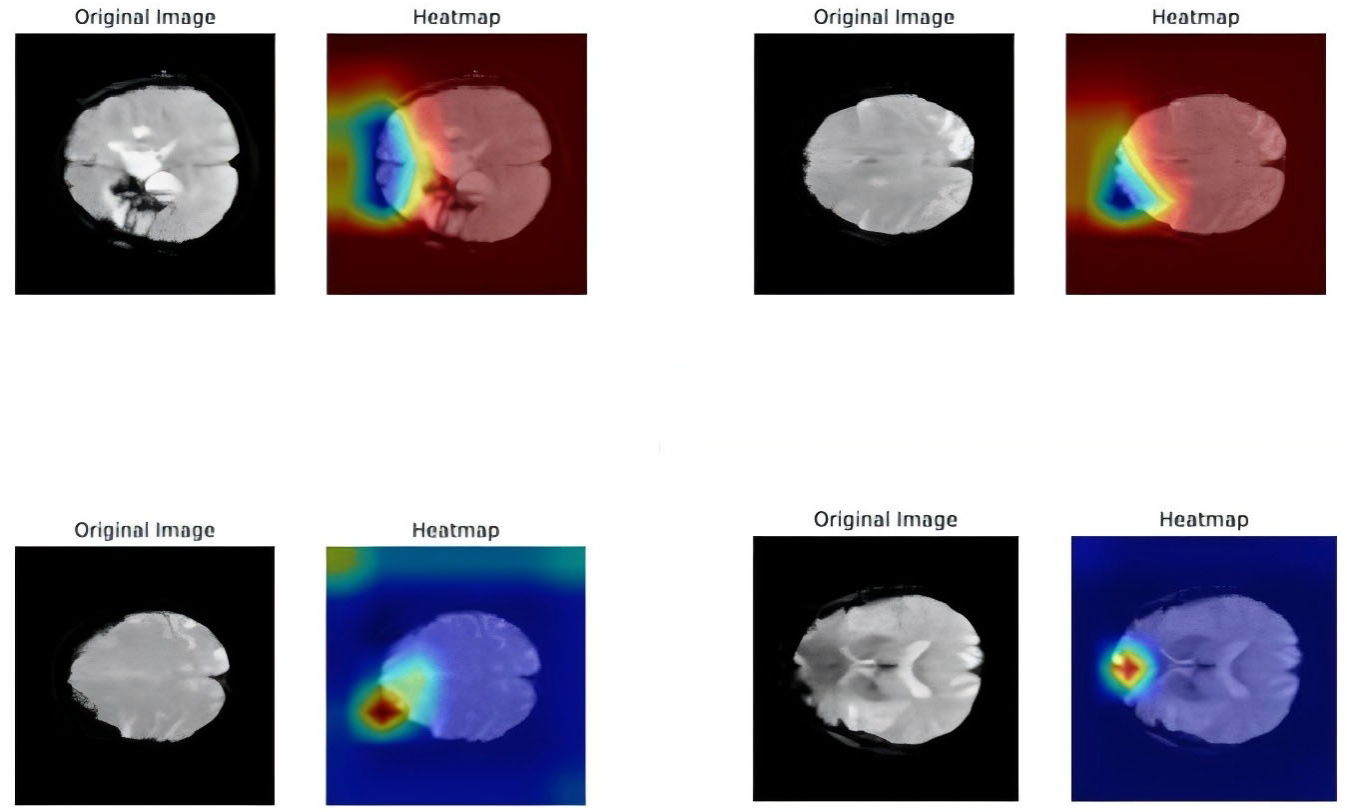
Gradient-weighted class activation mapping heatmap. The left is the original fMRI image, and the right is the heat map superimposed onto the original fMRI section.

In this study, feature maps in the middle layer of the model were weighted according to specific rules. The heatmap visualization enables an assessment of which input regions receive higher weight or “attention” when the model makes predictions or classifications. A color gradient typically indicates weight magnitude, with darker colors representing greater attention. The fMRI slice presented in Figure 2, where the Grad-CAM attention map was generated, is an axial view at the level of the frontal motor cortex. Specifically, this slice corresponds to the anatomical plane encompassing the primary motor cortex (M1) and the supplementary motor area (SMA) within the frontal lobe–regions critically involved in the regulation of lower-limb motor function. As highlighted in the heatmap, regions of high attention overlap with the lateral and medial frontal gyri, which is consistent with their established roles in post-stroke motor recovery, as demonstrated in previous neuroimaging studies. These findings suggest that, in the context of rehabilitation outcome prediction, the model places significant emphasis on image features derived from motor-related cortical regions, indicating a potential functional correlation with the rehabilitation process.

## 4 Discussion

Research on predicting motor function recovery following ischemic stroke often begins with an analysis of basic clinical data. Although clinical factors alone contribute to stroke rehabilitation predictions, their predictive accuracy remains limited. In recent years, various methods for forecasting functional recovery after stroke have gained significant research attention (Liang et al., 2022). Deep learning techniques utilize large datasets to train and develop models capable of handling new data, (LeCun et al., 2015; Sarker, 2021) demonstrating success across various domains. The clinical condition of ischemic stroke patients generates extensive information and image data (González, 2012).

Deep neural networks are developed by adjusting parameters such as the loss function, learning rate, and iterations. The optimal model is then selected based on accuracy evaluations and comparisons (Fan et al., 2022). Multi-layered complex networks effectively capture intricate relationships between input and output variables (Kammeraad et al., 2020). CNNs are a subset of deep neural networks designed to process two-dimensional data with multiple channels. These networks repeatedly apply convolution and pooling operations to extract meaningful features from input data, (Hwang et al., 2021) making them effective for image pattern recognition and processing. CNN-based deep learning methods have been utilized to develop EEG models capable of transforming EEG data into motor function scores (Li et al., 2022). In Kim et al. (2021) developed a CNN model for patients with coronary radiation-induced infarction. By extracting three consecutive T2-weighted brain MRI images at the level of the lateral ventricles for each patient, the model was trained to predict independent gait recovery 6 months post-infarction.

This study aims to predict rehabilitation outcomes in stroke patients 3 months post-event by developing a deep learning multimodal fusion prediction model. A comparative analysis of five models–FBNet, GhostNet, RegNet, ShuffleNet, and ResNet50–reveals that FBNet outperforms all others across evaluation metrics, demonstrating strong classification capabilities. These findings indicate that FBNet not only predicts categories with greater accuracy but also effectively covers samples from different classes. Across all metrics, GhostNet scores slightly lower, particularly in terms of its F1-score, which is limited to 0.6264. This suggests some imbalance between precision and recall, potentially influenced by data distribution or the adaptability of the pre-trained model, warranting further investigation in future experiments.

RegNet exhibits the weakest performance among the models, despite achieving a relatively high precision of 0.7595. However, its recall is limited to 0.6667, resulting in a low overall F1-score of 0.5917. This indicates difficulty in correctly identifying positive class samples, although for categories it can predict, accuracy remains relatively high. The model’s limitations may be attributed to lower complexity or restricted feature extraction capabilities, which hinder its ability to capture data characteristics in specific scenarios.

In the electroencephalogram and electromyogram group, the LSTM model was applied for classification analysis of sequential data. Overall, the LSTM model demonstrated stable performance in processing electroencephalogram and electromyogram sequential data. Although its metrics are slightly lower than those of the top-performing image models, such as FBNet and ShuffleNet, its classification ability remains strong, considering it relies solely on sequential data. These results suggest that electroencephalogram and electromyogram data provide useful information for classification tasks; however, some degree of information loss or noise may be present, limiting the model’s overall performance.

In the multimodal fusion group, classification performance improved significantly by integrating the outputs of the image model and the LSTM sequence model. The FBNet_LSTM fusion model achieved a validation accuracy of 0.8 with an F1-score of 0.7741, ranking as the second-best among the fusion models. A further enhancement was observed with the ShuffleNet_LSTM fusion model, which achieved an accuracy of 0.8031 and an F1-score of 0.7829, highlighting the advantages of multimodal fusion in complementing information. This fusion approach enables the spatial features of image data and the temporal features of sequence data to be jointly leveraged, allowing for a more comprehensive understanding of data patterns.

In contrast, the fusion models of GhostNet_LSTM and RegNet_LSTM achieved validation accuracies of 0.7754 and 0.76, respectively, surpassing their individual image models. However, their overall performance remained slightly lower than that of FBNet_LSTM and ShuffleNet_LSTM. This suggests that the inherent limitations of GhostNet and RegNet in feature extraction may have influenced the final fusion outcome. Additionally, although the fusion model of ResNet50_LSTM showed some improvement, its accuracy and F1-score remained significantly lower than those of other fusion models. This may be attributed to the weak performance of ResNet50 in the standalone image classification task. It can be inferred that weaker unimodal models may not fully exploit the potential of the data, even when incorporated into a multimodal fusion framework.

A comprehensive analysis indicates that multimodal fusion is an effective approach for enhancing classification performance. By integrating data from different modalities, the strengths of individual features can be better utilized. ShuffleNet_LSTM demonstrated the highest performance within the multimodal fusion group, confirming the synergistic effect of the lightweight model and the sequence network. FBNet_LSTM ranked as the second-best fusion model, further emphasizing the significance of FBNet’s image feature extraction capabilities within the fusion framework. Overall, multimodal fusion emerges as the optimal strategy for performance improvement in this task, and further exploration of more advanced fusion mechanisms may enhance the results even further.

Due to the inherent black-box nature of deep learning models, visualizing the complex computational processes and decision mechanisms during inference remains challenging. Consequently, an increasing number of studies have focused on enhancing the interpretability of deep learning models (Pan et al., 2023). Given the complexity of brain structure and function, conventional data visualization methods often fail to effectively illustrate the decision-making basis of these models. The gradient-weighted class activation map provides valuable insights into model behavior by highlighting the regions of high activation. As shown in the figure, these activated brain regions indicate that the model heavily relies on their image features for rehabilitation prediction, suggesting a strong correlation with the rehabilitation process, which has been clinically validated. Model visualization not only allows researchers to analyze the internal mechanisms of deep learning models, facilitating improvements in model architecture and parameter optimization, but also provides clinicians with visualization tools to accurately identify rehabilitation intervention targets, thereby accelerating patient recovery.

Resting-state functional magnetic resonance imaging (fMRI) offers a macroscopic view of brain network connectivity (e.g., motor network integrity), while simultaneous electroencephalogram (EEG) and electromyography (EMG) capture micro-level cortical-muscular dynamics, such as the latency between motor cortex activation and peripheral muscle response. Combined, these modalities provide complementary neurophysiological information essential for predicting rehabilitation outcomes. The proposed ShuffleNet-LSTM model addresses the limitations of unimodal approaches by integrating spatial resolution of fMRI with the temporal sensitivity of EEG/EMG, thereby enhancing predictive performance. This multimodal synergy accounts for the model’s superior classification accuracy (AUC: 0.8665), compared to models based solely on fMRI (AUC: 0.7169) or EEG/EMG (AUC: 0.6800). Clinically, the model offers the following utilities:(1) early identification of patients at risk of poor recovery within 2 weeks post-stroke, enabling timely initiation of intensive rehabilitation; (2) stratification of patients based on predicted recovery trajectories to optimize resource allocation; (3) individualized rehabilitation planning through interpretation of Grad-CAM attention maps.

## 5 Limitations and prospects

This study adopts a binary outcome framework for predictive analysis, primarily to address the urgent clinical demand for rapid prognosis assessment. This categorical approach offers practical utility by providing clear guidance for acute-phase treatment planning and prioritization of rehabilitation resources. However, we acknowledge that treating the lower-limb Fugl-Meyer Assessment (FMA) score as a continuous variable would yield more granular information. Continuous outcomes not only quantify degrees of functional recovery but also capture subtle changes in motor performance over time, thereby offering more refined insights for adjusting individualized rehabilitation strategies. Therefore, future research will focus on continuous outcome prediction as a key direction. We plan to incorporate model architectures such as deep neural networks (DNNs), which are well-suited for regression tasks. By integrating larger longitudinal datasets–currently being collected–that capture dynamic changes in FMA scores across multiple time points, we aim to develop dynamic regression models capable of phase-specific tracking and predictive optimization of the rehabilitation process.

Furthermore, the relatively small sample size of this study may limit the model’s generalizability, particularly in capturing rehabilitation heterogeneity among ischemic stroke subtypes (such as different infarction locations and disease duration). Future multi-center collaborative studies will incorporate larger patient cohorts with broader clinical characteristics (including comorbidities and diverse rehabilitation interventions) to enhance the model’s adaptability to varied clinical scenarios and improve its clinical reliability. This study has certain limitations, including a small sample size. Future research will focus on expanding the study through multicenter trials to enhance the generalizability and robustness of the findings.

## 6 Conclusion

The ShuffleNet_LSTM model achieved the highest performance among the multimodal fusion models, confirming the synergistic effect of the lightweight model and the sequence network. This combination significantly enhances the accuracy of long-term lower limb motor function state prediction and dynamic prognosis. The results provide strong technical support for precise evaluation and data-driven decision-making in related fields, contributing to improved rehabilitation strategies and clinical assessments.

## Data Availability

The raw data supporting the conclusions of this article will be made available by the authors, without undue reservation.
